# Supplementary material for: Generic comparison of lumen nucleation and fusion in epithelial organoids with and without hydrostatic pressure
Source: Nat Commun. 2025 Jul 8;16:6307. doi: 10.1038/s41467-025-60780-8 (PMC12238380; doi:10.1038/s41467-025-60780-8)
Supplement: Supplementary file 2 — Description of Additional Supplementary Files [file 41467_2025_60780_MOESM2_ESM.pdf]

Description of Additional Supplementary Files

Movies Captions.

Supplementary Movie 1 - Lumen nucleation after cell division for MDCK cyst. MDCK cells expressing E-cadherin in green and Podocalyxin. Time in hh:mm:ss.

Supplementary Movie 2 - Lumen nucleation after cells meet for MDCK cyst. MDCK cells expressing E-cadherin in green and Podocalyxin. Time in hh:mm:ss.

Supplementary Movie 3 - Lumen nucleation after cell division for pancreatic sphere. Time in hh:mm.

Supplementary Movie 4 - Lumen nucleation after cells meet for pancreatic sphere. Time in hh:mm.

Supplementary Movie 5 - Lumen increase in volume leads to lumen fusion for MDCK cyst. MDCK cells expressing E-cadherin in green and Podocalyxin in red with 8 cells as initial conditions. Time in hh:mm.

Supplementary Movie 6 - Lumen increase in volume leads to lumen fusion for pancreatic spheres. Pancreatic spheres were imaged with 8 cells as initial condition. Time in hh:mm.

Supplementary Movie 7 - Centripetal motion with low lumen occupancy leads to lumen fusion in epiblast. Time in hh:mm.

Supplementary Movie 8 - Simulation movie of typical numerical evolution of cysts.

Supplementary Movie 9 - Simulation movie of lumen interaction and fusion for 8 cells condition – with a dynamic similar to MDCK cyst.

Supplementary Movie 10 - Simulation movie of lumen interaction and fusion for 8 cells – with a dynamic similar to pancreatic sphere.

Supplementary Movie 11 - Simulation movie of lumen fusion for 8 cells condition – with a dynamic similar to epiblast.
